# Evaluation of the effects of erythropoietin and interleukin-6 in rats submitted to acute spinal cord injury

**DOI:** 10.6061/clinics/2019/e674

**Published:** 2019-08-13

**Authors:** Alderico Girão Campos de Barros, Alexandre Fogaça Cristante, Gustavo Bispo dos Santos, Renato José Mendonça Natalino, Ricardo José Rodriguez Ferreira, Tarcísio Eloy Pessoa de Barros-Filho

**Affiliations:** I Instituto Nacional de Ortopedia e Traumatologia (INTO), Rio de Janeiro, RJ, BR; II Instituto de Ortopedia e Traumatologia (IOT), Hospital das Clinicas HCFMUSP, Faculdade de Medicina, Universidade de Sao Paulo, Sao Paulo, SP, BR

**Keywords:** Interleukin-6, Erythropoietin, Spinal Cord Injuries, Central Nervous System/Injuries, Rats

## Abstract

**OBJECTIVE:**

To evaluate the effects of interleukin-6 (IL-6) and erythropoietin (EPO) in experimental acute spinal cord injury (SCI) in rats.

**METHODS:**

Using standardized equipment, namely, a New York University (NYU) Impactor, a SCI was produced in 50 Wistar rats using a 10-g weight drop from a 12.5-mm height. The rats were divided into the following 5 groups of 10 animals each: “Group EPO”, treated with erythropoietin only; “Group EPO + IL-6”, treated with both substances; “Group IL-6”, receiving IL-6 administration only; “Group Placebo”, receiving a placebo solution; and “Group Sham”, submitted to an incomplete procedure (only laminectomy, without SCI). All drugs and the placebo solution were administered intraperitoneally for three weeks. The animals were followed up for 42 days. Functional motor recovery was monitored by the Basso, Beattie, and Bresnahan (BBB) scale on days 2, 7, 14, 21, 28, 35 and 42. Motor-evoked potential tests were performed on the 42nd day. Histological analysis was performed after euthanasia.

**RESULTS:**

The group receiving EPO exhibited superior functional motor results on the BBB scale. IL-6 administration alone was not superior to the placebo treatment, and the IL-6 combination with EPO yielded worse results than did EPO alone.

**CONCLUSIONS:**

Using EPO after acute SCI in rats yielded benefits in functional recovery. The combination of EPO and IL-6 showed benefits, but with inferior results compared to those of isolated EPO; moreover, isolated use of IL-6 resulted in no benefit.

## INTRODUCTION

The devastating deficits and disability caused by spinal cord injuries (SCIs) are the target of researchers worldwide who seek pharmaceutical, surgical or mechanical treatment options 1 because this type of trauma most often affects productive workers 2. The pharmacological treatments explored in experimental and clinical trials are usually aimed at avoiding the secondary medullary injury that follows the initial mechanical trauma 1. High doses of methylprednisolone have been criticized recently for their limited evidence of benefit 3-5. Corticoids seem to cause important side effects 3,6,7. Laboratories have been trying to fill the gap in providing drugs such as estrogen, progesterone, erythropoietin, and magnesium for further testing in clinical trials 5,8.

Erythropoietin hormone plays a role in red cell homeostasis and seems to improve metabolism in many tissues 9. In rabbits 10 and rats 11,12, erythropoietin has shown a protective effect against experimental ischemic spinal injury. It likely reduces cell death by apoptosis and regulates the inflammatory activity of cytokines 13, thereby promoting angiogenesis 14, reestablishing vascular autoregulation 15-17 and reducing lipid peroxidation 18. Significantly better functional results have been obtained with erythropoietin compared to the control treatment in a recent experimental trial 19, which, although appearing weeks after the treatment started, allowed the authors to suggest that there might be a possible mitigating effect of the hormone on secondary SCI.

In addition to erythropoietin, other molecular mediators of neuroaxonal growth and regeneration that have been the focus of most studies are interleukins (ILs) 1, 2, 4, 6 and 10 and interferon-gamma 18,20,21. The roles of these T-cell cytokines in injury-induced neural damage and repair are complex, and their interaction has been investigated 22. Researchers have been trying to obtain better axonal regeneration without excessive scarring, which could impair axon growth. The use of interleukin in low dosages can result in less scarring and better axonal regeneration 23, and IL-6 acts directly on Schwann cells (apparently the highest source of Il-6 24), releasing glial fibrillary acid protein (GFAP), which is necessary for neuronal regeneration 25.

While the results with erythropoietin seem promising, there is still some controversy about the role of cytokines as a therapeutic approach in SCI 26. Therefore, we aimed to evaluate the functional, histological, and motor-evoked potential (MEP) effects of both agents (namely, erythropoietin and IL-6) on the treatment of SCI in rats. The hypothesis was that there would be a synergistic effect between erythropoietin and IL-6 that could lead to neurological improvement better than that yielded by the isolated drugs.

## METHODS

### Study design and ethics

This was an experimental study with rats, conducted in the same university laboratory specialized in spinal cord trauma as that in previous studies 19,27,28. The present investigation followed all national and international guidelines and regulations for animal experimentation and pain control. Furthermore, the university's ethics committee approved the study protocol.

In this study, we divided rats into five groups of ten animals each. All rats in four of the groups underwent experimental SCI as described below, and each group received one type of treatment or placebo. The fifth group underwent laminectomy only, without SCI (the sham group).

### Sample size and allocation

We based the sample size of 50 rats on other experiments with spinal cord lesions using the same method proposed here. This study randomized 50 rats into groups considering possible losses during follow-up 8,19,27-32. A computer sequence generator randomized the allocation. Rats had their tails marked with different codes according to the allocation group. After the experimental and therapeutic procedures, we evaluated them for function, and the evaluators were blind to the animal allocation because they did not know the meaning of the tail codes. After euthanasia, a pathologist also blinded to the animal allocation and the other evaluations performed histological analyses of the spinal cord tissue.

We divided rats into the following five groups:

- **“Group EPO”** - undergoing a spinal cord lesion and receiving 1,000 IU/kg of body weight of erythropoietin (EPO) intraperitoneally, daily for three weeks;

- **“Group EPO + IL-6”** - undergoing a spinal cord lesion and receiving the same dose of EPO plus 200 UI/100 g of interleukin-6 (IL-6) intraperitoneally, daily for three weeks;

- **“Group IL-6”** - undergoing a spinal cord lesion and receiving only IL-6 at the same dose above, during the same time;

- **“Group Placebo”** - undergoing spinal cord lesion and receiving saline intraperitoneally daily for three weeks;

- **“Group Sham”** - undergoing laminectomy only, without a spinal cord lesion or any therapeutic procedure.

We administered the drugs immediately after the spinal cord contusion. After the experiments, the rats remained in the cages for 48 hours and then underwent a functional evaluation using the Basso, Beattie, and Bresnahan (BBB) 33 scale at seven specific time points, an MEP exam (on day 42 after the lesion) and a histological evaluation after euthanasia.

### Animal care and pain control

In this study, we used male, young adult Wistar rats weighing 340 to 450 g, all from the same bioterium. The rats were all similar regarding size, weight and health conditions, and all had a good health status and healthy skin on visual inspection. Furthermore, they all presented normal motricity on the baseline (21 points in the BBB scale).

Animals were stimulated to move before the experiment so that they became accustomed to the handling by researchers. They were maintained in cages measuring 60 cm x 40 cm, up to five animals each cage, with water and feeding *ad libitum*. The laboratory had a controlled temperature (25°C).

We excluded rats from the study if they had a persistent infection (present after 10 days of antibiotic therapy), if they lost more than 10% of their body weight after the spinal cord lesion, if they died immediately after the spinal cord lesion, or if the lesion was not effective (and the rat had normal motricity after the lesion). Autophagia or mutilation behaviors during the follow-up period were also exclusion criteria.

Rats underwent spinal cord lesions under anesthesia using 100 mg/kg of ketamine and 5 mg/kg of xylazine. For local anesthesia, we used lidocaine hydrochloride with epinephrine. Anesthesia was confirmed by the absence of corneal and tail reflexes and hind paw reactions at compression 34. The anesthetic agents took effect after 5 min and lasted for at least 2 h. For MEP evaluations, we administered pentobarbital (55-75 mg/kg) intraperitoneally and used the lethal dose of 140 mg/kg, followed by potassium chloride intravenously for euthanasia, on day 42.

After laminectomy, spinal cord lesioning and suturing, the animals received an antibiotic (cephazolin, 2 mg/100 g intraperitoneally, single dose) and pain medication (tramadol, hydrochloride, 5 mg/100 g intramuscularly for five days and meloxicam, 2 mg/kg, once daily for seven days). The bladder of each rat was emptied by manual pressure daily. Blood detected in the urine was an indication for levofloxacin use (2.5 mg/100 g for 10 days) due to urinary infection. Persistent infection after 10 days was an indication for study exclusion and immediate euthanasia to prevent transmission to other rats.

### Experimental procedures

Once anesthetized, the animals were trichotomized in the dorsal region for the laminectomy procedure, according to the methods standardized in the laboratory 28,30-32,34. An incision was made in the skin, following the medial dorsal line, reaching the aponeurotic and muscular planes, and exposing the posterior vertebral arches from T8 to T12, with subperiosteal dissection of spinous processes and laminae from T9 to T11. Hemostasis was performed using a bipolar coagulator when needed. The spinous process and laminae of the T10 vertebra were removed using a bone rongeur, together with the distal half of the T9 spinous process, so that the spinal cord was exposed. This procedure allowed the positioning of the tip of an NYU Impactor device for experimental spinal cord lesions. The NYU Weight-Drop Impactor (New York University Medical Center, New York, NY, USA) 33 was used to produce a controlled moderate spinal cord lesion at the thoracic level (T8-T11) in all rats, except those in Group Sham.

A drop of a 10-g rod from a height of 12.5 mm was used to produce a moderate spinal cord lesion, as described previously 8,19,28. We then washed the lesion site with saline and sutured the muscles, fascia, and skin using nylon monofilaments (2.0).

### Functional evaluation

Two trained researchers simultaneously evaluated motricity using the BBB scale, which ranges from zero (no movement) to 21 points (normal motricity), and they were blind to each other's scoring and to the animal allocation. When there was disagreement in their registries, we recorded the lowest score for analysis. This evaluation with the BBB scale took place on days 2, 7, 14, 21, 28, 35 and 42 after the spinal cord lesion.

### Neurophysiological evaluation

The MEP exam was used to evaluate the muscle response to electrical stimulation 42 days after the spinal cord lesion. Under the anesthetic procedure described above, the rat was shaved, and electrodes were positioned in the head and limbs as described previously 18,35. The electrodes had paired needles with a fixed distance, and the muscles chosen were the distal extensors of the anterior and posterior limbs. The following parameters were used for the records: SWEEP SPEED: 2.0 ms/div and GAIN: 2 mV. All MEP exams were conducted by the same evaluator, who was blind to the animal allocation. Latency and amplitude values were recorded for each rat.

### Histological evaluation

The animals were sacrificed after the MEP exam, and spinal cords were removed from C3 to T10 for histological evaluation. The tissues were identified and fixed in 10% formalin, and the 2-mm fragments were bathed in alcohol, diaphanized in xylol and embedded in liquid paraffin. The paraffin blocks were then cut into 5-μm-thick sections using a Leica microtome (MR 2055, Wetzlar, Germany). The sections were placed on glass slides, bathed in saline and stained with hematoxylin and eosin.

The same pathologist evaluated all slides for necrosis, hemorrhage, hyperemia, nerve degeneration and cellular infiltrates, according to the following scale: zero, indicating absence; 1 point, representing a mild presence; 2 points, indicating a moderate presence; and 3 points, designating a strong presence of the feature. This pathologist was blind to the group allocation.

The slides were also fixed in osmium tetroxide solution and stained with 2% toluidine blue at 1% to allow the regenerated axon neuron count. The pathologist chose two areas with a good representation of cells from each section (cranial and caudal, with 40x magnification) and considered neurons with diameters greater than 15 μm for counting. The software Sigma Scan Pro 5.0 (Sigma, San Jose, CA, USA) was used for neuron counting.

### Outcomes and statistical analysis

The primary outcome evaluated in this study was the BBB score on day 42. MEP amplitude and latency results and histological results (scoring and neuron counts) were considered secondary outcomes. As the results had a Gaussian distribution, we used parametric and nonparametric tests. We employed analysis of variance (ANOVA) or the Kruskal-Wallis test for the comparison of groups. Student's t and Mann-Whitney tests with Bonferroni correction were used for comparisons between groups. For the paired comparison over time, repeated measures ANOVA or the Friedman test was employed.

We used the software SPSS 20.0 for Mac for the statistical analysis and considered a difference as significant when type I error was equal to or less than 0.05.

## RESULTS

During the six weeks of the study, all rats were equally taken care of according to the protocol described above, with pain control and antibiotic therapy. Nevertheless, four rats died. In Group EPO, rat #8 died of infection in the third week, and rat #10 was excluded in the fourth week due to autophagic behavior. In Group EPO + IL-6, we had to exclude rat #5 for autophagy in the sixth week. In Group Placebo, rat #9 died of an infection in the third week. In Group IL-6 and Group Sham, there was no death.

### Functional analysis

Table 1 presents a descriptive analysis of the BBB scores. The difference between the moments of the evaluation was significant for all five groups (*p*=0.001 for Group EPO and *p*=0.0001 for the others; Friedman test). Because it did not suffer SCI, Group Sham received a score of 21 in all evaluations (indicating the maximum score and normal function).

**Figure 1:**
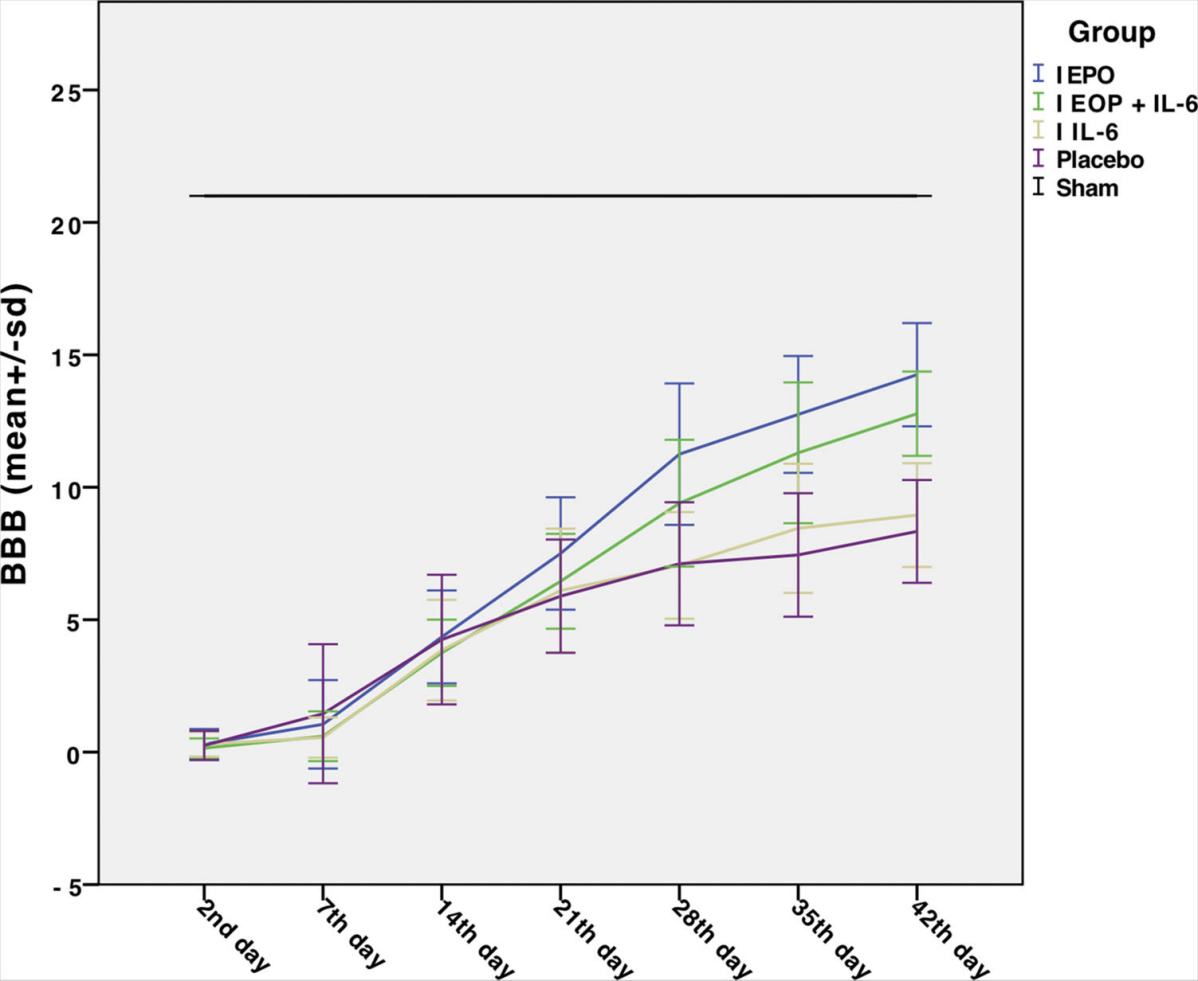
Evolution of the scores on the Basso, Beattie and Bresnahan (BBB) motor scale of the five groups over the seven (2^nd^, 7^th^, 14^th^, 21^st^, 28^th^, 35^th^ and 42^nd^ days) evaluation periods. The blue line indicates the results of Group EPO, the green line indicates the evolution of Group EPO + IL-6, the orange line indicates the evolution of Group IL-6, and the purple line indicates Group Placebo. The black line represents Group Sham, which did not suffer spinal cord injury (SCI) and therefore remained unchanged with respect to function.

Figure 1 illustrates the evolution of BBB scores of the five groups over the seven (2^nd^, 7^th^, 14^th^, 21^st^, 28^th^, 35^th^ and 42^nd^ day) assessment periods. There was a significant difference between Group EPO and Group EPO + IL-6 (*p*<0.001). Additionally, there was a significant difference between Group EPO + IL-6 and Group IL-6 (*p*<0.0001). Although it seemed more important for Group IL-6 than for Group Placebo, this difference was not statistically significant (*p*>1). The values of the BBB scale in Group Sham were constant, as expected.

### Evoked potential analysis

**Figure 2:**
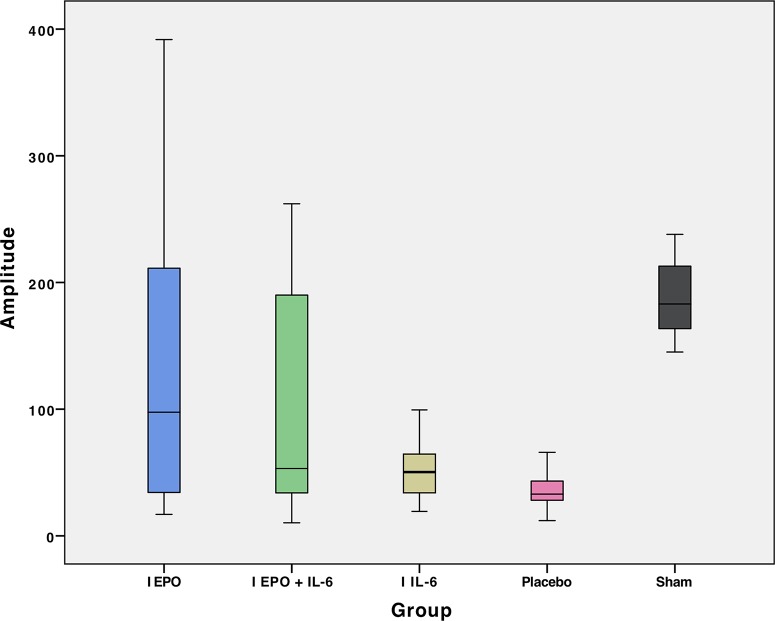
Mean amplitude in the motor-evoked potential (MEP) exam of hindlimbs for each group.

**Figure 3:**
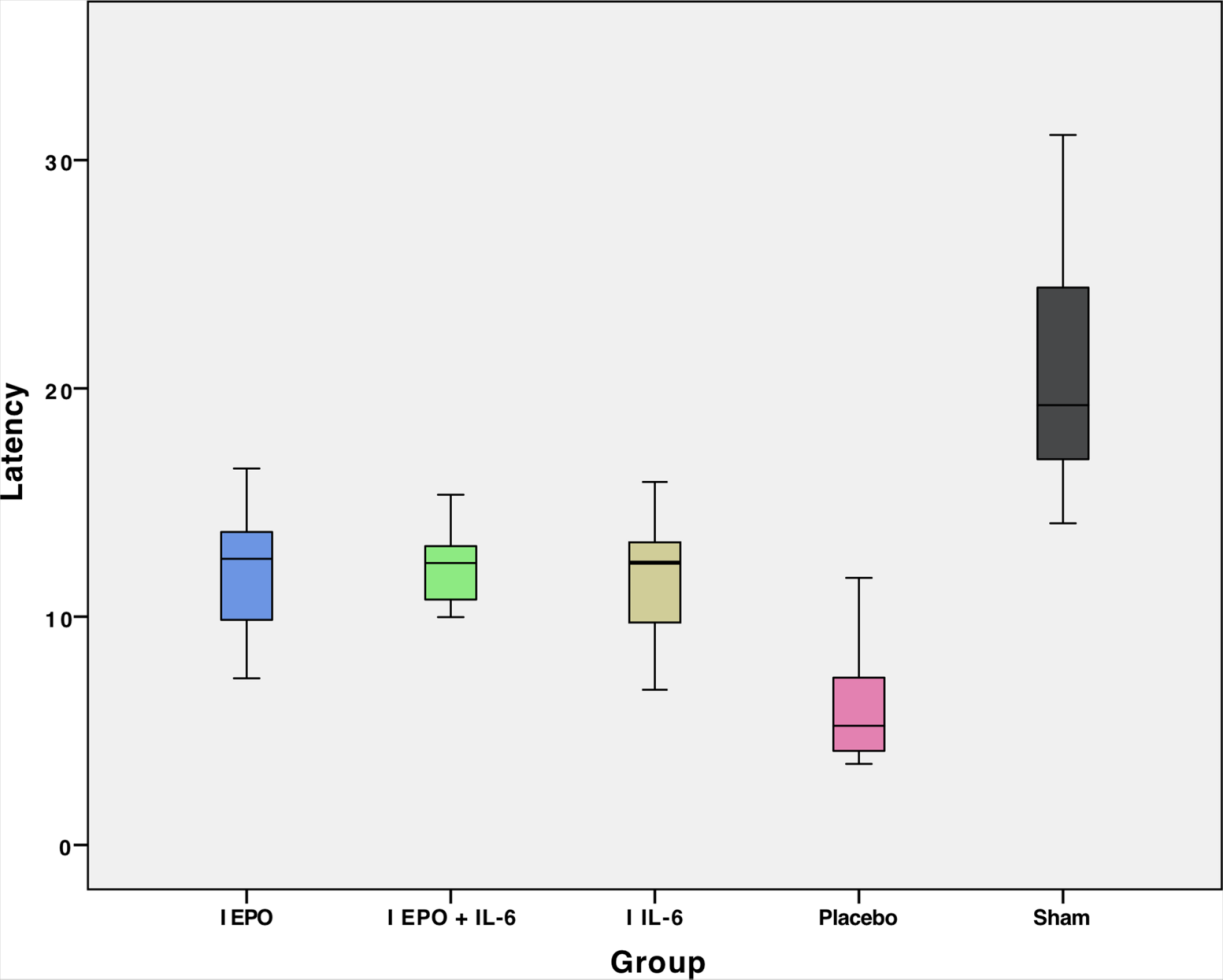
Mean latency in the motor-evoked potential (MEP) exam of hindlimbs for each group.

The results of the MEP evaluation in 46 rats are shown in Table 2, containing the mean and standard deviation values of latency and amplitude. The mean values of amplitude and latency per group are presented in Figures 2 and 3, respectively. Table 3 compares the mean amplitude and latency values, according to the *p*-values, by group.

### Histological analysis

There were statistically significant differences (Kruskal-Wallis test) among the five groups for all histological variables (necrosis, hemorrhage, hyperemia, degeneration and infiltrate), except for hemorrhage. As the Kruskal-Wallis test showed a difference among all groups altogether, we applied the Mann-Whitney test for pairwise comparisons. Table 4 shows these results. Groups EPO and EPO + IL-6 were similar. Likewise, the neuron count analysis showed no significant differences between groups (Bonferroni test), except for obvious differences from the Sham group.

## DISCUSSION

In our study, compared to treatment with placebo or EPO, the administration of IL-6 after acute SCI did not yield any significant differences in neurological recovery. The association of EPO with IL-6 seemed to have a negative effect on the EPO action since the analyses showed that EPO was better than EPO plus IL-6 and that this combination was better than IL-6 or placebo.

IL-6 plays a key role in traumatic central nervous system (CNS) injuries, and it is one of the most common proinflammatory cytokines in the acute and subacute phases after trauma to the spinal cord, actively participating in secondary injury to the spinal nervous tissue 36. The occurrence of an injury is accompanied by a substantial increase in the concentration of IL-6 and other proinflammatory cytokines. This elevation starts approximately 30 minutes after the trauma and lasts for approximately five hours. After this period, there was a decline in the expression of these mediators before the appearance of macrophages and neutrophils 24 hours after the trauma. This early peak suggests that IL-6 and other proinflammatory cytokines are mainly produced by cells of the CNS itself (microglia and neurons) rather than by infiltrating leukocytes as originally thought 37. Experimental studies corroborate this theory, showing that in the presence of a traumatic CNS injury, the IL-6 receptor is present in oligodendrocytes and neurons and that IL-6-induced neuritis promotes increased neuronal survival by inducing the production of neurotrophic factors 38,39. These pieces of evidence prompted us to investigate IL-6 as a possible pharmacological treatment in SCI.

However, the role of IL-6 in the inflammatory response after SCI is still not well explained. Perhaps this interleukin functions as both a neurotrophic factor and a cytotoxic factor. There are some explanations for these seemingly contradictory functions. This initial elevation in the expression of IL-6 and other inflammatory cytokines in the first hours after SCI may be damaging due to overproduction of these mediators, and there is a rather narrow window for the difference between benefit and toxicity 40. *In vitro* studies have shown that cells need to be intact, without traumatic injury, to produce the IL-6 neurotrophic effects, and this situation may not occur in vivo 37. In fact, the cellular activities of cytokines are complex and subject to variations in concentration, receptors, subtypes and time of administration 41,42. Studies show that the injection of cytokines into the spinal cord four days after injury promotes a decrease in the activation of microglia, a reduction in the recruitment of circulating macrophages and tissue loss. However, when these same cytokines are given one day after the injury, the posttraumatic inflammatory response and tissue loss are better 43,44.

To evaluate tissue characteristics, we included a histopathological analysis of the spinal cord of the animals with SCI in our study to assess the degree of cellular damage and evidence of neuronal morphological alterations. However, previous experiences in our laboratory have suggested that hematoxylin and eosin staining for histological evaluation of the spinal cord may be not sensitive enough to detect significant differences. This is why we opted to evaluate neuronal cell counting also, but in our study, there was no significant difference in cell counts between groups. One of the possible explanations for this finding is the choice to induce a moderate lesion in this experimental study 45: we used a height of 12.5 mm for the weight drop on the spinal cord of the rats. This choice prioritized functional changes more than morphostructural changes. Therefore, functional analyses were our priority.

The functional analysis using the BBB scale showed that the rats submitted to treatment with erythropoietin (Group EPO) presented significantly better results than the rats from other groups at the end of the six weeks. In the neurophysiological analysis through the MEP, there was also no significant difference in the mean values of amplitude between Groups EPO and EPO + IL-6. Group Sham had the highest mean latency, which we considered to be counterintuitive because the animals in this group did not suffer SCI and presented a normal score on the BBB functional scale. We did not take into consideration the mean latency results obtained because the literature shows that this parameter can be influenced by the small size of the animal, and even a minimal amount of preserved nerve fibers can transmit the electric impulse, biasing the interpretation 46.

EPO has a neuroprotective role in the ischemic and traumatic lesions of the CNS, as demonstrated in experimental trials 10-12. It acts by reducing the inflammatory response, promoting angiogenesis, inhibiting lipid peroxidation and modulating the antiapoptotic response 12,47,48. In our study, over the weeks, Group EPO presented higher scores in the evaluation by the BBB scale. This improvement allows us to strengthen the hypothesis of the role of EPO as a neuroprotective agent 46. We believe that the use of EPO in the treatment of acute SCI should be further investigated and that other variables, such as dose, time of treatment, route of administration, synergism with other drugs, and clinical applicability should be addressed.

The results of the use of erythropoietin in acute medullary lesions in rats are also conflicting in the literature 19,46,49. This controversy may perhaps be explained by differences in study designs, pertaining to the dose, time of treatment, animal race or strain and patterns of the medullary lesion. Additionally, some mechanisms of erythropoietin action are still unknown. We can infer that the results of Group EPO + IL-6 were inferior to those of Group EPO because the presence of IL-6 negatively modified the action of EPO. As previously mentioned, interleukins have dynamic effects that change between neuroprotection and neurotoxicity. These complex and unknown mechanisms were not the focus of this study. However, our results allow us to suppose that these variations may be very subtle because although the BBB scores for Group EPO + IL-6 were lower than the scores for Group EPO, the BBB scores in Group IL-6 were not inferior to those of Group Placebo. This finding suggests that there may be some antagonism between IL-6 and EPO, rather than neurotoxicity. Additionally, the histological evaluation and the MEP exams did not show any significant differences between Groups EPO and EPO + IL-6. Further studies are needed to clarify these gaps better.

## CONCLUSION

The administration of erythropoietin after experimental SCI in rats showed benefits for motor recovery. The combination of erythropoietin and IL-6 yielded benefits, but with lower results than erythropoietin alone. Isolated use of IL-6 showed no benefit after experimental SCI in rats.

## AUTHOR CONTRIBUTIONS

Barros AGC and Cristante AF designed the study, performed experiments and collected data, interpreted results e wrote the manuscript. Santos GB, Natalino RJM and Ferreira RJR collected data, interpreted results and critically reviewed the manuscript. Barros-Filho TEP collaborated in the study design and critical review of the manuscript. All authors reviewed and approved the final version of the manuscript to be published.

## Figures and Tables

**Table 1:** Mean and standard deviation (SD) for the Basso, Beattie and Bresnahan (BBB) scores (mean and SD) in each group in the seven moments of evaluation.

	Group	Mean	SD
2^nd^ day	EPO	0.375	0.619
	EPO + IL-6	0.167	0.383
	IL-6	0.300	0.470
	Placebo	0.278	0.574
	Sham	21	0
7^th^ day	EPO	1.063	1.878
	EPO + IL-6	0.444	0.511
	IL-6	0.550	0.759
	Placebo	1.611	2.725
	Sham	21	0
14^th^ day	EPO	4.688	1.815
	EPO + IL-6	3.611	1.195
	IL-6	3.850	1.899
	Placebo	4.722	2.080
	Sham	21	0
21^st^ day	EPO	7.563	2.250
	EPO + IL-6	6.444	1.885
	IL-6	6.100	2.337
	Placebo	5.889	2.139
	Sham	21	0
28^th^ day	EPO	11.250	2.670
	EPO + IL-6	9.667	2.376
	IL-6	7.050	2.012
	Placebo	7.111	2.323
	Sham	21	0
35^th^ day	EPO	12.750	2.206
	EPO + IL-6	11.667	2.543
	IL-6	8.450	2.438
	Placebo	7.444	2.331
	Sham	21	0
42^nd^ day	EPO	14.250	1.949
	EPO + IL-6	12.778	1.592
	IL-6	8.950	1.959
	Placebo	8.333	1.940
	Sham	21	0

**Table 2:** Motor-evoked potential (MEP) results: means and standard deviations (SDs) for latency and amplitude for the hindlimbs of the animals in each group.

	Group	Mean	SD
Latency	EPO	12.038	2.72
	EPO + IL-6	12.135	1.62
	IL-6	11.842	2.25
	Placebo	6.108	2.33
	Sham	20.791	4.93
Amplitude	EPO	126.858	26.18
	EPO + IL-6	103.959	20.66
	IL-6	49.356	4.41
	Placebo	34.413	3.09
	Sham	189.064	6.76

**Table 3:** Comparison of mean latency (LAT) and mean amplitude (AMP) in the motor-evoked potential (MEP) exam: *p*-values.

Group	*p*: AMP mean (mA)	*p*: LAT mean (ms)
EPO *versus* EPO + IL-6	<1.000	<1.000
EPO *versus* IL-6	<0.003	<1.000
EPO *versus* Placebo	<0.001	<0.001
EPO *versus* Sham	<0.031	<0.001
EPO + IL-6 *versus* IL-6	<0.071	<1.000
EPO + IL-6 *versus* Placebo	<0.009	<0.001
EPO + IL-6 *versus* Sham	<0.001	<0.001
IL-6 *versus* Placebo	<1.000	<0.001
IL-6 *versus* Sham	<0.001	<0.001
Placebo *versus* Sham	<0.001	<0.001

**Table 4:** Pairwise group comparison of the results from the histological analysis: *p*-values.

	*p* values				
Groups	Necrosis	Bleeding	Hyperemia	Degeneration	Infiltrate
EPO *versus* EPO + IL-6	0.114	0.071	0.536	0.963	0.423
EPO *versus* IL-6	0.408	0.536	0.161	0.055	0.965
EPO *versus* Placebo	0.236	0.918	0.758	0.481	0.541
EPO *versus* Sham	**0.016**	0.364	**0.002**	**0.001**	0.173
EPO + IL-6 *versus* IL-6	0.053	0.315	0.447	**0.028**	0.400
EPO + IL-6 *versus* Placebo	0.063	0.222	0.666	0.387	0.094
EPO + IL-6 *versus* Sham	0.661	0.356	**0.0001**	**0.0001**	0.447
IL-6 *versus* Placebo	0.720	0.780	0.133	0.156	0.549
IL-6 *versus* Sham	**0.004**	0.853	**0.0001**	**0.0001**	0.143
Placebo *versus* Sham	**0.006**	0.604	**0.0001**	**0.0001**	**0.017**

***p*<0.05.**
